# Weighted Sum Secrecy Rate Maximization for Joint ITS- and IRS-Empowered System

**DOI:** 10.3390/e25071102

**Published:** 2023-07-24

**Authors:** Shaochuan Yang, Kaizhi Huang, Hehao Niu, Yi Wang, Zheng Chu

**Affiliations:** 1 Wireless Communication Technology Office, Information Engineering University, Zhengzhou 450001, China; yangsc@zua.edu.cn; 2 School of Intelligent Engineering, Zhengzhou University of Aeronautics, Zhengzhou 450046, China; yiwang@zua.edu.cn; 3 Sixty-Third Research Institute, National University of Defense Technology, Nanjing 210007, China; niuhaonupt@foxmail.com; 4 5GIC & 6GIC, Institute for Communication Systems (ICS), University of Surrey, Guildford GU2 7XH, UK; andrew.chuzheng7@gmail.com

**Keywords:** intelligent reflecting surface (IRS), intelligent transmission surface (ITS), alternating direction method of multiplier (ADMM), majorization–minimization (MM) algorithm, physical layer security (PLS)

## Abstract

In this work, we investigate a novel intelligent surface-assisted multiuser multiple-input single-output multiple-eavesdropper (MU-MISOME) secure communication network where an intelligent reflecting surface (IRS) is deployed to enhance the secrecy performance and an intelligent transmission surface (ITS)-based transmitter is utilized to perform energy-efficient beamforming. A weighted sum secrecy rate (WSSR) maximization problem is developed by jointly optimizing transmit power allocation, ITS beamforming, and IRS phase shift. To solve this problem, we transform the objective function into an approximated concave form by using the successive convex approximation (SCA) technique. Then, we propose an efficient alternating optimization (AO) algorithm to solve the reformulated problem in an iterative way, where Karush–Kuhn–Tucker (KKT) conditions, the alternating direction method of the multiplier (ADMM), and majorization–minimization (MM) methods are adopted to derive the closed-form solution for each subproblem. Finally, simulation results are given to verify the convergence and secrecy performance of the proposed schemes.

## 1. Introduction

With the development of Internet of Things (IoT) systems, a massive number of data are transmitted via wireless networks, which are very vulnerable to eavesdropping attacks due to the broadcast nature of the wireless medium. Information security has become a non-negligible issue in sixth-generation (6G) networks. Physical layer security (PLS) is a promising technique to enhance system security via exploiting the inherent characteristics of the wireless channel. The theoretical basis of PLS comes from Shannon’s information-theoretic secrecy research [1]. Then, Wyner introduced the wiretap channel model and derived secrecy capacity in degraded broadcast channels [2]. Afterwards, the idea of PLS was extended to non-degraded broadcast channels [3], Gaussian channels [4], multi-antenna channels [5], and so on. Nowadays, various schemes, such as artificial noise (AN), beamforming, and cooperative relaying, have been utilized to improve the secrecy rate [6]. However, these techniques increase the computational complexity and hardware cost, which have become challenging issues with a massive number of devices in 6G networks. Moreover, when the channel responses of the legitimate receivers and the eavesdroppers are highly correlated, traditional schemes also benefit eavesdroppers [7].

Recently, intelligent surfaces have been proposed as a potential alternative technique for building an energy-efficient wireless networks [8]. To be specific, an intelligent surface is a two-dimensional surface consisting of many low-cost, passive, reconfigurable elements. The intelligent surface consumes much less power than conventional antennas since these elements require no dedicated radio frequency (RF) chains or amplifiers [9]. Each element of an intelligent surface can dynamically adjust the amplitude and/or phase of the incident electromagnetic (EM) wave, which enables the intelligent surface to actively customize wireless propagation environments [10]. Moreover, intelligent surfaces can be flexibly deployed on building surfaces without introducing additional interference. Depending on the operation mode of the intelligent surface elements, an intelligent surfaces is mainly divided into two categories: an intelligent reflecting surface (IRS) and an intelligent transmission surface (ITS).

Many studies have been carried out to examine IRS-aided PLS. For instance, Ref. [11] proposed a power-efficient beamforming scheme for IRS-aided multi-antenna secure transmission where a closed-form expression of the optimal beamformer is derived. In [12], the achievable secrecy rate maximization problem of the IRS-aided multiuser (MU) system is alternatively optimized by a block coordinate decent (BCD) algorithm. An IRS-aided secrecy simultaneous wireless information and power transfer (SWIPT) network was considered in [13], where a penalty dual decomposition (PDD)-based algorithm was proposed to solve a max–min fairness robust problem. Ref. [14] focused on the secrecy performance of IRS-aided massive MIMO systems with statistical channel state information (CSI), where an approximate expression of the sum achievable security data rate was derived.

The above works mainly focus on adopting intelligent surfaces as auxiliary nodes. Actually, apart from being deployed as a passive reflector, intelligent surfaces can also be equipped at the base station (BS) serving as an antenna array. Without the requirement for a conventional RF combiner and phase shifter, intelligent surface-based transmitters consume significantly less power than conventional transmitters [15]. Because of advantages including higher aperture efficiency, larger working bandwidth, and no feed occlusion or self-interference, the ITS-based transmitter is more promising than the IRS-based transmitter in complicated communication environments [16]. In Ref. [17], the author proposed an ITS-based transmitter in the MISO channel, where difference-of-convex (DC) programming was used to maximize the achievable sum-rate. Further, the authors of [18] investigated the SWIPT networks with ITS-based transmitters, where a robust transmission scheme was proposed in the case of imperfect CSI. In Ref. [19], a novel joint intelligent surface-assisted secure network was investigated for the first time, where PDD and element-wise Lagrange dual methods were utilized to design ITS beamforming vector and IRS phase shift.

It can be seen that existing studies primarily focused on the standalone use of intelligent surfaces as additional nodes or antenna arrays. Based on the above analysis, we consider secure transmission over multiple-input single-output multiple-eavesdropper (MISOME) channels where an ITS-aided transmitter and an IRS are jointly deployed to improve system secure performance. Specifically, we constructed a WSSR maximization problem by optimizing transmit power allocation, the ITS beamforming vector, and the IRS phase shift vector. Since the original problem is non-concave, we first use successive convex approximation (SCA) to approximate the object function by its linear lower bound. Then, we decompose the reformulated problem into three subproblems through the AO technique, where the transmit power allocation optimization subproblem is solved iteratively by using Karush–Kuhn–Tucker (KKT) conditions, and the optimization subproblem of ITS beamforming vector and IRS phase shift vector are solved by the ADMM and MM methods, respectively. The main contributions of this article are summarized as follows:

(1) We propose a novel intelligent surface-assisted MU-MISOME network architecture, where an ITS-aided transmitter sends a confidential signal to several legitimate users with the assistance of an IRS in the presence of multiple eavesdroppers. Specifically, the ITS reduces the power consumption and hardware cost of the transmitter by replacing power amplifiers and the RF chains. Moreover, the IRS is utilized to configure the radio propagation environment aiming for efficiently enlarging the channel difference between Bobs and Eves. Furthermore, we derived the WSSR to characterize the secure performance of the system.

(2) We propose an energy-efficient transmission scheme to maximize the WSSR by jointly designing transmit power allocation, ITS beamforming, and the IRS phase shift. As the objective function is non-concave and the optimization variables are highly coupled, we firstly transform the objective function into a more feasible concave form and solve it alternatively via the AO algorithm. Specifically, we derive the analytical solution for transmit power allocation by employing the KKT conditions, while the ITS beamforming and IRS phase shift are kept fixed. To solve the ITS beamforming and IRS phase shift optimization subproblem, the ADMM and MM methods are proposed.

(3) The simulation results validate the accuracy of our derived results and show the effectiveness of the proposed algorithms.

The rest of this paper is organized as follows. In Section 2, we present a MU-MISOME downlink secure transmission network assisted by an ITS-based transmitter and IRS. In Section 3, the WSSR maximization problem is reformulated and solved by an AO algorithm. Our theoretical analysis is verified by simulation results in Section 4, and conclusions are drawn in Section 5.

*Notation*: Column vectors and matrices are represented as boldface lower-case and upper-case letters, respectively. The transpose, conjugate, and conjugate transpose of A are represented, respectively, as $A^{T}$, $A^{*}$, and $A^{H}$. A≻0 represents that A is a Hermitian positive definite matrix. $ℜ\left\{a\right\}$, $\left|a\right|$, and ∠a denote the real part, the absolute value, and the angle of a complex value *a*, respectively. CN(0,δ) represents a circularly symmetric complex Gaussian (CSCG) random variable with zero mean and covariance δ. The letter *j* stands for the imaginary unit $\sqrt{-1}$.

## 2. System Model and Signal Representation

**Figure 1:**
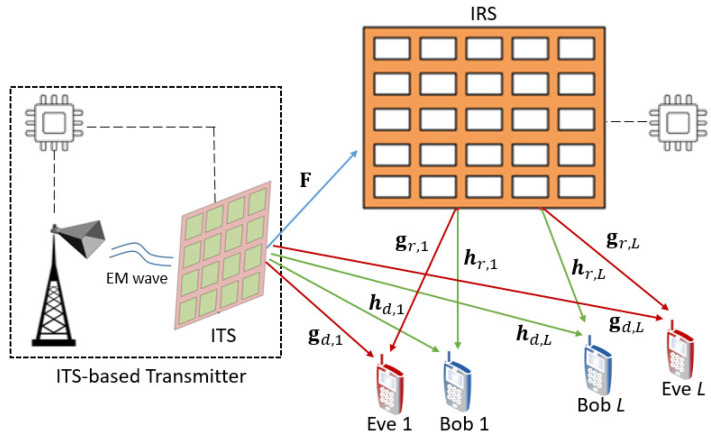
System model.

In this paper, we consider a secure MU-MISOME downlink broadcast system as illustrated in Figure 1. An ITS-based transmitter (Alice) intends to send *L* independent confidential data streams for each of the *L* legitimate users (Bobs) over the same frequency band simultaneously in the presence of *L* internal untrusted non-cooperative eavesdroppers (Eves) that are arbitrarily distributed around Bobs. An IRS is employed to assist the secure communication. All Bobs and Eves are equipped with a single antenna, and the number of transmissive elements at ITS and that of reflective elements at IRS are denoted as *N* and *M*, respectively. Similar to [20], the CSI of all links is assumed to be perfectly known at the transmitter, and the IRS that makes the results presented in this paper can be regarded as the performance upper bound of the considered system. For convenience, we summarize the key notations utilized throughout this paper in Table 1.

### 2.1. Channel Model

The channel coefficients from Alice to the IRS, from Alice to the *l*th Bob/Eve, and from the IRS to the *l*th Bob/Eve (*l* = 1, …, *L*) are denoted, respectively, by $F\in C^{M\times N}$, $h_{d,l}\in C^{N\times 1}$; $g_{d,l}\in C^{N\times 1}$; $h_{r,l}\in C^{M\times 1}$; and $g_{r,l}\in C^{M\times 1}$. Without a loss of generality, we assume that $g_{d,l}$ and $h_{d,l}$ experience the Rayleigh fading. However, the IRS-related wireless channels are modeled as Rician fading due to the fact that the IRS is commonly deployed on the high-rise buildings near the desired receivers. Thus, we have

(1) $$h=\sqrt{\frac{\kappa_{h}}{\kappa_{h}+1}}h^{LoS}+\sqrt{\frac{1}{\kappa_{h}+1}}h^{NLoS}$$

where $h\in H=\left\{F,h_{r,l},g_{r,l}\right\}$; $\kappa_{h}$ is the Rician factor; and $h^{LoS}$ and $h^{NLoS}$ represent line-of-sight (LoS) and non-LoS (NLoS) components of channel h, respectively. The NLoS components $h^{NLoS}$ follow the Rayleigh fading. The LoS components $h^{LoS}$ is given by $h^{LoS}=a_{r}{\left(a_{t}\right)}^{H}$, where $a_{t}$ and $a_{r}$ are the array response vectors of the corresponding transmitter and receiver, respectively. In this paper, we assume both IRS and ITS are modeled as the uniform planar array (UPA). For a H×V UPA, the array response vector is formulated as follows:

(2) $$\begin{array}{c} a\left(\nu ,\psi \right)=\frac{1}{\sqrt{HV}}\left[1,\ldots ,e^{j2\pi \frac{d_{e}}{\lambda }\left(m\sin\left(\nu \right)\sin\left(\psi \right)+n\cos\left(\psi \right)\right)},\right.\ldots ,{\left.e^{j2\pi \frac{d_{e}}{\lambda }\left(\left(H-1\right)\sin\left(\nu \right)\sin\left(\psi \right)+\left(V-1\right)\cos\left(\psi \right)\right)}\right]}^{T} \end{array}$$

where $d_{e}$ represents the spacing between two adjacent array elements; λ is the carrier wavelength; and ν and ψ denote the azimuth and elevation angle of arrival (or departure), respectively. $m\in \left[0,H\right]$ and $n\in \left[0,V\right]$ represent the horizontal and vertical element indices at the array, respectively.

### 2.2. Signal Model for ITS and IRS

The ITS-based transmitter we considered consists of a feed antenna and an ITS. The feed antenna sends a single frequency electromagnetic (EM) wave to the ITS. To facilitate the analysis, the EM wave emitted from the feed antenna is assumed to be transmitted through ITS completely [18]. The ITS performs signal modulation and beamforming on the incident wave by adjusting the amplitude and phase of each transmissive element [17]. In this work, we focus on the beamforming design of ITS, and the beamforming vector is denoted by $w\;=\;{\left[w_{1},\ldots ,w_{N}\right]}^{T}\in C^{N\times 1}$, where $w_{n}\in W\overset{\Delta }{=}\left\{w_{n}\left|w_{n}=\alpha_{n}e^{j\beta_{n}},\alpha_{n}\in \left[0,1\right],\beta_{n}\in \left[0,2\pi \right),\forall n\right.\right\}$, $\alpha_{n}$, and $\beta_{n}$ represent the amplitude and phase response of the *n*th transmissive element, respectively. Similarly, we let $\theta ={\left[\theta_{1},...,\theta_{M}\right]}^{T}\in C^{M\times 1}$ denote the phase shift vector of the IRS where $\theta_{m}\in X\overset{\Delta }{=}\left\{\theta_{m}\left|\theta_{m}=e^{j\phi_{m}},\phi_{m}\in \left[0,2\pi \right),\forall m\right.\right\}$, $\phi_{m}$ denotes the phase shift of the *m*th reflective element of the IRS. Note that the amplitude of IRS phase shift is normalized, i.e., $\left|\theta_{m}\right|=1,\forall m$ because each IRS reflective element only modifies the phase of the incident wave without changing amplitude. Moreover, we ignore the signal reflected multiple times as a result of severe path loss [20].

### 2.3. Signal Transmission Model

The confidential message transmitted to the *l*th Bob is denoted as $s_{l}$, which is assumed as the i.i.d CSCG random variable, i.e., $s_{l}∼\;CN\left(0,1\right),\forall l$. Then, the signal sent by Alice can be formulated as $x=w\sum_{l=1}^{L}a_{l}s_{l}$, where $a_{l}$ is the corresponding power allocation factor for $s_{l}$. Thus, the received signal at the *l*th Bob/Eve can be represented as

(3) $$\begin{array}{c} y_{d,l}=\left(h_{d,l}^{H}+h_{r,l}^{H}\Theta^{H}F\right)w\sum_{i=1}^{L}a_{i}s_{i}+n_{d,l} \end{array}$$

(4) $$\begin{array}{c} y_{e,l}=\left(g_{d,l}^{H}+g_{r,l}^{H}\Theta^{H}F\right)w\sum_{i=1}^{L}a_{i}s_{i}+n_{e,l} \end{array}$$

respectively, where $\Theta =diag\left(\theta_{1},...,\theta_{M}\right)$ is the phase shift matrix of IRS. $n_{d,l}∼CN\left(0,\sigma_{d,l}^{2}\right)$ and $n_{e,l}∼CN\left(0,\sigma_{e,l}^{2}\right)$ represent additive white Gaussian noise at the *l*th Bob/Eve, respectively. By letting $\hat{\theta }={\left[\theta^{H},1\right]}^{H}$, $H_{l}={\left[\mathrm{diag}\left(h_{r,l}^{H}\right)F,h_{d,l}^{H}\right]}^{T}$, and $G_{l}={\left[\mathrm{diag}\left(g_{r,l}^{H}\right)F,g_{d,l}^{H}\right]}^{T}$, the received signals in (3) and (4) can be reformulated as follows:

(5) $$\begin{array}{c} y_{d,l}={\hat{\theta }}^{H}H_{l}w\sum_{i=1}^{L}a_{i}s_{i}+n_{d,l} \end{array}$$

(6) $$\begin{array}{c} y_{e,l}={\hat{\theta }}^{H}G_{l}w\sum_{i=1}^{L}a_{i}s_{i}+n_{e,l} \end{array}$$

Assuming each Eve only attempts to eavesdrop its nearest Bob, the received signal-to-interference-plus noise ratio (SINR) at the *l*th Bob/Eve can be written as [19]

(7) $$\begin{array}{c} r_{d,l}=\frac{{\left|{\hat{\theta }}^{H}{\tilde{H}}_{l}wa_{l}\right|}^{2}}{\sum_{i=1,i\neq l}^{L}{\left|{\hat{\theta }}^{H}{\tilde{H}}_{l}wa_{i}\right|}^{2}+1} \end{array}$$

(8) $$\begin{array}{c} r_{e,l}=\frac{{\left|{\hat{\theta }}^{H}{\tilde{G}}_{l}wa_{l}\right|}^{2}}{\sum_{i=1,i\neq l}^{L}{\left|{\hat{\theta }}^{H}{\tilde{G}}_{l}wa_{i}\right|}^{2}+1} \end{array}$$

respectively, where ${\tilde{H}}_{l}=H_{l}/\sigma_{d,l}$, ${\tilde{G}}_{l}=G_{l}/\sigma_{e,l}$. The achievable secrecy rate for the *l*th Bob in bits/second/Hertz (bps/Hz) can be given as

(9) $$R_{s,l}={\left[R_{d,l}-R_{e,l}\right]}^{+}$$

where ${\left[z\right]}^{+}\overset{\Delta }{=}\max\left(z,0\right)$, $R_{d,l}=\ln\left(1+r_{d,l}\right)$, and $R_{e,l}=\ln\left(1+r_{e,l}\right)$ denote the achievable rates of the *l*th Bob/Eve, respectively. Since the optimal value of our problem must be non-negative, the operator ${\left[\cdot \right]}^{+}$ is omitted for simplicity in the remaining parts of this paper.

## 3. Problem Formulation and Solution for Wssr Maximization

In this work, our objective is to jointly optimize the power allocation ${\left\{a_{l}\right\}}_{l=1}^{L}$, the ITS transmission coefficient w, and the IRS phase shift $\hat{\theta }$ to maximize the WSSR of the system. Let $\eta_{l}$ denote the weight for the *l*th Bob, which is used to represent the priority of the *l*th Bob in the system. Thus, the WSSR maximization problem can be generally formulated as

(10a) $$\begin{array}{cc} \max_{{\left\{a_{l}\right\}}_{l=1}^{L},w,\hat{\theta }} & R_{s}\overset{\Delta }{=}\sum_{l=1}^{L}\eta_{l}\left[\ln\left(1+r_{d,l}\right)-\ln\left(1+r_{e,l}\right)\right] \end{array}$$

(10b) $$\begin{array}{cc} \; & s.t.\sum_{l=1}^{L}a_{l}^{2}\leq P_{s},a_{l}\geq 0,\forall l \end{array}$$

(10c) $$\begin{array}{cc} \; & w_{n}\in W,\forall n \end{array}$$

(10d) $$\begin{array}{cc} \; & \theta_{m}\in X,\forall m,{\hat{\theta }}_{M+1}=1 \end{array}$$

where $R_{s}$ represents WSSR of the system; constraint (10b) defines the total transmit power constraint; and constraints (10c,d) characterize the constraints of the ITS beamforming and the phase shifts of the IRS, respectively. The proposed WSSR maximization problem (10) is challenging to solve directly, mainly because of the non-concave objective function as well as the coupling relation of the optimization variables. In order to tackle this, we first approximately reformulate the objective function (10a) into a tractable form by deriving its concave lower bound. Then, we decouple the optimization variables and decompose the transformed problem into three solvable subproblems by utilizing the alternating optimization (AO) technique, and each subproblem is efficiently solved via the iterative algorithm. Finally, we present the overall algorithm and analyze its convergence and complexity

### 3.1. Problem Transformation

To start with, let $\left\{{\left\{a_{l}^{t}\right\}}_{l=1}^{L},w^{t},{\hat{\theta }}^{t}\right\}$ denote a given point in problem (10) at the *t*th iteration and introduce the following lemma.

**Lemma** **1** ([21]). *For any u and v>0, we have*

(11) $$\ln\left(1+\frac{{\left|u\right|}^{2}}{v}\right)\geq \ln\left(1+\frac{{\left|u^{t}\right|}^{2}}{v^{t}}\right)-\frac{{\left|u^{t}\right|}^{2}}{v^{t}}+\frac{2ℜ\left\{{\left(u^{t}\right)}^{*}u\right\}}{v^{t}}-\frac{{\left|u^{t}\right|}^{2}\left(v+{\left|u\right|}^{2}\right)}{v^{t}\left(v^{t}+{\left|u^{t}\right|}^{2}\right)}$$

*where $\left\{u^{t},v^{t}\right\}$ are fixed points.*

Based on Lemma 1, we can find a lower bound of $R_{d,l}$ and an upper bound of $R_{e,l}$ around $\left\{{\left\{a_{l}^{t}\right\}}_{l=1}^{L},w^{t},{\hat{\theta }}^{t}\right\}$, which can be expressed as

(12) $$R_{d,l}\geq \ln\left(1+\frac{{\left|x_{l}^{t}\right|}^{2}}{y_{l}^{t}}\right)-\frac{{\left|x_{l}^{t}\right|}^{2}}{y_{l}^{t}}+\frac{2ℜ\left\{{\left(x_{l}^{t}\right)}^{*}x_{l}\right\}}{y_{l}^{t}}-\frac{{\left|x_{l}^{t}\right|}^{2}\left(y_{l}+{\left|x_{l}\right|}^{2}\right)}{y_{l}^{t}\left(y_{l}^{t}+{\left|x_{l}^{t}\right|}^{2}\right)}\overset{\Delta }{=}R_{d,l}^{lb}$$

(13) $$\begin{array}{c} \begin{array}{c} R_{e,l}\leq \ln\left(1+z_{l}^{t}\right)+\frac{1+z_{l}}{1+z_{l}^{t}}-\ln\left(1+c_{l}^{t}\right)+c_{l}^{t}\\ -2\sum_{i=1,i\neq l}^{L}ℜ\left\{a_{i}{\left(w\right)}^{H}{\tilde{G}}_{l}^{H}\hat{\theta }{\left({\hat{\theta }}^{t}\right)}^{H}{\tilde{G}}_{l}w^{t}a_{i}^{t}\right\}\\ +\frac{c_{l}^{t}}{1+c_{l}^{t}}\left(1+\sum_{i=1,i\neq l}^{L}{\left|{\hat{\theta }}^{H}{\tilde{G}}_{l}wa_{i}\right|}^{2}\right)-1\overset{\Delta }{=}R_{e,l}^{ub} \end{array} \end{array}$$

respectively, where $x_{l}={\hat{\theta }}^{H}{\tilde{H}}_{l}wa_{l}$, $x_{l}^{t}={\left({\hat{\theta }}^{t}\right)}^{H}{\tilde{H}}_{l}w^{t}a_{i}^{t}$, $y_{l}=\sum_{i=1,i\neq l}^{L}{\left|{\hat{\theta }}^{H}{\tilde{H}}_{l}wa_{i}\right|}^{2}+1$, $y_{l}^{t}=\sum_{i=1,i\neq l}^{L}{\left|{\left({\hat{\theta }}^{t}\right)}^{H}{\tilde{H}}_{l}w^{t}a_{i}^{t}\right|}^{2}+1$, $z_{l}=\sum_{i=1}^{L}{\left|{\hat{\theta }}^{H}{\tilde{G}}_{l}wa_{i}\right|}^{2}$, $z_{l}^{t}=\sum_{i=1}^{L}{\left|{\left({\hat{\theta }}^{t}\right)}^{H}{\tilde{G}}_{l}w^{t}a_{i}^{t}\right|}^{2}$, and $c_{l}^{t}=\sum_{i=1,i\neq l}^{L}{\left|{\left({\hat{\theta }}^{t}\right)}^{H}{\tilde{G}}_{l}w^{t}a_{i}^{t}\right|}^{2}$.

**Proof.**  Please refer to Appendix A.    □

By substituting (12) and (13) into (10a) and neglecting the constant terms, we transform the problem (10) into a tractable approximated form around the fixed point $\left\{{\left\{a_{l}^{t}\right\}}_{l=1}^{L},w^{t},{\hat{\theta }}^{t}\right\}$ as follows:

(14a) $$\begin{array}{cc} \min_{{\left\{a_{l}\right\}}_{l=1}^{L},w,\hat{\theta }} & \sum_{l=1}^{L}\eta_{l}\left\{\frac{c_{l}^{t}}{1+c_{l}^{t}}\left[\sum_{i=1,i\neq l}^{L}{\left|{\hat{\theta }}^{H}{\tilde{G}}_{l}wa_{i}\right|}^{2}\right]+\frac{{\left|x_{l}^{t}\right|}^{2}\sum_{i=1}^{L}{\left|{\hat{\theta }}^{H}{\tilde{H}}_{l}wa_{i}\right|}^{2}}{y_{l}^{t}\left(y_{l}^{t}+{\left|x_{l}^{t}\right|}^{2}\right)}+\frac{\sum_{i=1}^{L}{\left|{\hat{\theta }}^{H}{\tilde{G}}_{l}wa_{i}\right|}^{2}}{1+z_{l}^{t}}\right.\\ \; & -\left.2\sum_{i=1,i\neq l}^{L}R\left\{a_{i}w^{H}{\tilde{G}}_{l}^{H}\hat{\theta }{\left({\hat{\theta }}^{t}\right)}^{H}{\tilde{G}}_{l}w^{t}a_{i}^{t}\right\}+\frac{2R\left\{a_{i}w^{H}{\tilde{H}}_{l}^{H}\hat{\theta }{\left({\hat{\theta }}^{t}\right)}^{H}{\tilde{H}}_{l}w^{t}a_{i}^{t}\right\}}{y_{l}^{t}}\right\} \end{array}$$

(14b) $$\begin{array}{cc} \; & s.t.\left(10b\right)-\left(10d\right) \end{array}$$

However, problem (14) is still difficult to solve directly due to the highly coupled optimization variables in (14a). In the following part, we use the AO technique to decompose problem (14) into three subproblems and alternately update ${\left\{a_{l}\right\}}_{l=1}^{L}$, w and $\hat{\theta }$ while keeping the other variables fixed.

### 3.2. Power Allocation Optimization

In this subsection, we consider the optimization of ${\left\{a_{l}\right\}}_{l=1}^{L}$ with the given w and $\hat{\theta }$. By omitting the irrelevant term, the WSSR maximization problem with respect to power allocation ${\left\{a_{l}\right\}}_{l=1}^{L}$ is reformulated as follows:

(15a) $$\begin{array}{cc} \min_{{\left\{a_{l}\right\}}_{l=1}^{L}} & \sum_{l=1}^{L}\left(T_{l}a_{l}^{2}-2t_{l}a_{l}\right) \end{array}$$

(15b) $$\begin{array}{cc} & s.t.\left(10b\right) \end{array}$$

where

(16) $$\begin{array}{cc} & T_{0}=\sum_{i=1}^{L}\frac{\eta_{i}c_{i}^{t}{\left|{\left({\hat{\theta }}^{t}\right)}^{H}{\tilde{G}}_{i}w^{t}\right|}^{2}}{1+c_{i}^{t}}+\sum_{i=1}^{L}\frac{\eta_{i}{\left|x_{i}^{t}\right|}^{2}{\left|{\left({\hat{\theta }}^{t}\right)}^{H}{\tilde{H}}_{i}w^{t}\right|}^{2}}{y_{i}^{t}\left(y_{i}^{t}+{\left|x_{i}^{t}\right|}^{2}\right)}+\sum_{i=1}^{L}\frac{\eta_{i}{\left|{\left({\hat{\theta }}^{t}\right)}^{H}{\tilde{G}}_{i}w^{t}\right|}^{2}}{1+z_{i}^{t}}\\ & T_{l}=T_{0}-\frac{\eta_{l}c_{l}^{t}{\left|{\left({\hat{\theta }}^{t}\right)}^{H}{\tilde{G}}_{l}w^{t}\right|}^{2}}{1+c_{l}^{t}}\\ & t_{l}=\sum_{i=1,i\neq l}^{L}\eta_{i}R\left\{a_{l}^{t}{\left(w^{t}\right)}^{H}{\tilde{G}}_{i}^{H}{\hat{\theta }}^{t}{\left({\hat{\theta }}^{t}\right)}^{H}{\tilde{G}}_{i}w^{t}\right\}+\frac{\eta_{l}R\left\{a_{l}^{t}{\left(w^{t}\right)}^{H}{\tilde{H}}_{l}^{H}{\hat{\theta }}^{t}{\left({\hat{\theta }}^{t}\right)}^{H}{\tilde{H}}_{l}w^{t}\right\}}{y_{l}^{t}} \end{array}$$

Note that problem (15) is a quadratically constrained quadratic programming (QCQP), which can be solved by using a convex optimization solver, e.g., CVX [22]. In order to further reduce computational complexity, we propose a more efficient method by solving KKT conditions. The KKT conditions of the problem (15) with respect to ${\left\{a_{l}\right\}}_{l=1}^{L}$ are given as follows:

(17) $$\begin{array}{c} \frac{\partial L\left({\left\{a_{l}\right\}}_{l=1}^{L},\lambda \right)}{\partial a_{l}}=0 \end{array}$$

(18) $$\begin{array}{c} \lambda \left(\sum_{l=1}^{L}a_{l}^{2}-P_{s}\right)=0 \end{array}$$

where $L\left({\left\{a_{l}\right\}}_{l=1}^{L},\lambda \right)=\sum_{l=1}^{L}\left(T_{l}a_{l}^{2}-2t_{l}a_{l}\right)+\lambda \left(\sum_{l=1}^{L}a_{l}^{2}-P_{max}\right)$ is the Lagrangian function and λ≥0 is the Lagrangian multiplier associated with the constraint (10b). By solving (17), we can derive power allocation $a_{l}\left(\lambda \right)$ as

(19) $$a_{l}\left(\lambda \right)=\frac{t_{l}}{T_{l}+\lambda },\forall l$$

The optimal $\lambda^{*}$ can be calculated by solving the second KKT condition (18). We search $\lambda^{*}$ by considering the following two cases:

(1) If $a_{l}\left(0\right)$ satisfies the power constraint, i.e., $\sum_{i=1}^{L}a_{l}^{2}\left(0\right)\leq P_{max}$, then $\lambda^{*}=0$, and the optimum power allocation is obtained by $a_{l}^{*}=a_{l}\left(0\right)$.

(2) Otherwise, the full power constraint $\sum_{i=1}^{L}a_{l}^{2}\left(\lambda \right)=P_{max}$ should be met. Given (19), we have $\sum_{i=1}^{L}t_{l}^{2}/{\left(T_{l}+\lambda \right)}^{2}\;=P_{max}$. Since $\sum_{i=1}^{L}t_{l}^{2}/{\left(T_{l}+\lambda \right)}^{2}\;$ decreases monotonically with respect to λ, the optimal $\lambda^{*}$ can be found efficiently by applying the bisection method. Moreover, since $T_{l}>0\;\forall l$, we can set an upper searching bound on λ by letting $T_{l}=0\;\forall l$; thus, the search interval is $\left[0,\sqrt{\sum_{i=1}^{L}t_{l}^{2}/P_{max}\;}\right]$.

The detailed steps for calculating ${\left\{a_{l}^{*}\right\}}_{l=1}^{L}$ and $\lambda^{*}$ are summarized in Algorithm 1.
**Algorithm 1** Power Allocation Optimization
1: **Initialization:** set the accuracy ε, and set the searching bounds $\lambda_{l}$ and $\lambda_{u}$;
2: **Calculate** ${\left\{a_{l}\left(0\right)\right\}}_{l=1}^{L}$ according to (19). **If**
$\sum_{i=1}^{L}a_{l}^{2}\left(0\right)\leq P_{max}$, **then**
$a_{l}^{*}=a_{l}\left(0\right)\forall l$, $\lambda^{*}=0$ and **terminate**; **otherwise**, move to step 3;
3: **Repeat**
4: **Calculate**
$\lambda =\left(\lambda_{l}+\lambda_{u}\right)/2\;$;
5: **Update**
${\left\{a_{l}\left(\lambda \right)\right\}}_{l=1}^{L}$ via (19);
6: **If**
$\sum_{i=1}^{L}a_{l}^{2}\left(\lambda \right)\leq P_{max}$ set $\lambda_{l}=\lambda$; **Otherwise**, **set**
$\lambda_{u}=\lambda$
**end if**;
7: **Until**
$\left|\lambda_{l}-\lambda_{u}\right|\leq \varepsilon$;
8: **Output**
$\left\{{\left\{a_{l}^{*}\right\}}_{l=1}^{L},\lambda^{*}\right\}$.


### 3.3. Optimization of ITS Beamforming

In this subsection, we attempt to optimize the ITS beamforming vector w with given ${\left\{a_{l}\right\}}_{l=1}^{L}$ and $\hat{\theta }$. With some manipulations, the problem (14) can be reformulated as follows:

(20a) $$\begin{array}{cc} \min_{w}\; & w^{H}\mathrm{Aw}-2ℜ\left\{w^{H}b\right\} \end{array}$$

(20b) $$\begin{array}{cc} \; & s.t.(10c) \end{array}$$

where $A=\sum_{l=1}^{L}\eta_{l}A_{l}$ and $b=\sum_{l=1}^{L}\eta_{l}b_{l}$. A and b are, respectively, denoted as

(21) $$\begin{array}{cc} A_{l} & =\frac{c_{l}^{t}\sum_{i=1,i\neq l}^{L}{\left(a_{i}^{t}\right)}^{2}{\tilde{G}}_{l}^{H}{\hat{\theta }}^{t}{\left({\hat{\theta }}^{t}\right)}^{H}{\tilde{G}}_{l}}{1+c_{l}^{t}}+\frac{{\left|x_{l}^{t}\right|}^{2}\sum_{i=1}^{L}{\left(a_{i}^{t}\right)}^{2}{\tilde{H}}_{l}^{H}{\hat{\theta }}^{t}{\left({\hat{\theta }}^{t}\right)}^{H}{\tilde{H}}_{l}}{y_{l}^{t}\left(y_{l}^{t}+{\left|x_{l}^{t}\right|}^{2}\right)}+\frac{\sum_{i=1}^{L}{\left(a_{i}^{t}\right)}^{2}{\tilde{G}}_{l}^{H}{\hat{\theta }}^{t}{\left({\hat{\theta }}^{t}\right)}^{H}{\tilde{G}}_{l}}{1+z_{l}^{t}}\\ b_{l} & =\sum_{i=1,i\neq l}^{L}{\tilde{G}}_{l}^{H}{\hat{\theta }}^{t}{\left({\hat{\theta }}^{t}\right)}^{H}{\tilde{G}}_{l}w^{t}{\left(a_{i}^{t}\right)}^{2}+\frac{{\tilde{H}}_{l}^{H}{\hat{\theta }}^{t}{\left({\hat{\theta }}^{t}\right)}^{H}{\tilde{H}}_{l}w^{t}{\left(a_{l}^{t}\right)}^{2}}{y_{l}^{t}} \end{array}$$

Problem (20) is a quadraitic programming problem that can be solved by using the CVX toolbox. Here, we propose a low-complexity algorithm where a closed-form solution is derived iteratively by using the ADMM method. First, let us reformulate problem (20) as follows:

(22a) $$\begin{array}{cc} \min_{r,w}\;r^{H}\mathrm{Ar} & -2ℜ\left\{r^{H}b\right\} \end{array}$$

(22b) $$\begin{array}{cc} s.t.\left|w_{n}\right| & \leq 1,\forall n\in M \end{array}$$

(22c) $$\begin{array}{cc} r & =w \end{array}$$

where $r\in C^{M\times 1}$ is a slack variable. Then, the augmented Lagrange function of (20a) can be written as

(23) $$\begin{array}{cc} L\left(r,w,p\right) & =r^{H}\mathrm{Ar}-2ℜ\left\{r^{H}b\right\}-ℜ\left\{p^{H}\left(r-w\right)\right\}+\frac{\rho }{2}{∥r-w∥}^{2} \end{array}$$

where $p\in C^{N\times 1}$ is the Lagrange multiplier with respect to the constraint (22b) and ρ≥0 is the penalty factor. By using the ADMM method, we have the following iterations:

(24a) $$\begin{array}{cc} r^{k+1} & =arg\min_{r}L\left(r^{k},w^{k},p^{k}\right) \end{array}$$

(24b) $$\begin{array}{cc} w^{k+1} & =arg\min_{\left|w_{n}\right|=1\forall n}L\left(r^{k+1},w^{k},p^{k}\right) \end{array}$$

(24c) $$\begin{array}{cc} p^{k+1} & =p^{k}-\rho \left(r^{k+1}-w^{k+1}\right) \end{array}$$

According to first-order optimality condition of (23), we have

(25) $$2Ar^{k+1}-2b-p^{k}-\rho \left(r^{k+1}-w^{k}\right)=0$$

Then, the solution for (24a) is derived as

(26) $$r^{k+1}={\left(\rho I+2A\right)}^{-1}\left(2b+\rho w^{k}+p^{k}\right)$$

Problem (24b) is equivalent to $\min_{\left|w_{n}\right|=1\forall n\in N}{∥w-\left(r^{k+1}-\rho^{-1}p^{k}\right)∥}^{2}$, which is a projection problem, and the closed-form solution is given as

(27) $$\begin{array}{c} {\left[w^{k+1}\right]}_{n}=\left\{\begin{array}{cc} {\left[r^{k+1}-\rho^{-1}p^{k}\right]}_{n}, & \mathrm{if}{\left[r^{k+1}-\rho^{-1}p^{k}\right]}_{n}\leq 1\\ \frac{{\left[r^{k+1}-\rho^{-1}p^{k}\right]}_{n}}{\left|{\left[r^{k+1}-\rho^{-1}p^{k}\right]}_{n}\right|}, & \mathrm{if}{\left[r^{k+1}-\rho^{-1}p^{k}\right]}_{n}>1 \end{array}\right. \end{array}$$

where ${\left[w\right]}_{n}$ denotes the *n*th element of w. Then, by exploiting (24c) and (25), we obtain that $p^{k+1}\;=2Ar^{k+1}-2b$. The detailed step of the ADMM algorithm is stated in Algorithm 2, and it guarantees to converge when the value of the penalty parameter ρ satisfies: ρI/2−A≻0 [23].
**Algorithm 2** The ADMM algorithm for problem (20)
1: **Initialization:** set the maximum iteration number *K*; the accuracy ε; a feasible point $\left\{{\left\{a_{l}^{0}\right\}}_{l=1}^{L},w^{0},{\hat{\theta }}^{0}\right\}$; and the penalty factor ρ, which satisfies ρI/2−A≻0;
2: **Repeat**
*k*
3: **Update**
$r^{k+1}$ according to (26);
4: **Update** $w^{k+1}$ according to (27);
6: **Update** $p^{k+1}=2Ar^{k+1}-2b$;
7: **Until**
fwk+1−fwkfwk+1−fwkfwk+1fwk+1≤ε or k>K;
8: **Output** $w^{*}$.


### 3.4. Optimization of IRS Phase Shift

In this subsection, we aim to optimize the IRS phase shift $\hat{\theta }$ with given ${\left\{a_{l}\right\}}_{l=1}^{L}$ and w. We first recast the problem (14) as follows:

(28a) $$\begin{array}{cc} \min_{\hat{\theta }} & \;{\hat{\theta }}^{H}\Omega \hat{\theta }-2ℜ\left\{{\hat{\theta }}^{H}\varphi \right\} \end{array}$$

(28b) $$\begin{array}{cc} \; & s.t.\left(10d\right) \end{array}$$

where $\Omega =\sum_{i=1}^{L}\eta_{i}\Omega_{i}$, $\varphi =\sum_{i=1}^{L}\eta_{i}\varphi_{i}$, $\Omega_{i}$ and $\varphi_{i}$ are denoted by

(29) $$\begin{array}{cc} \Omega_{l} & =\frac{c_{l}^{t}}{1+c_{l}^{t}}\sum_{i=1,i\neq l}^{L}{\left(a_{i}^{t}\right)}^{2}{\tilde{G}}_{l}w^{t}{\left(w^{t}\right)}^{H}{\tilde{G}}_{l}^{H}+\frac{{\left|x_{l}^{t}\right|}^{2}\sum_{i=1}^{L}{\left(a_{i}^{t}\right)}^{2}{\tilde{H}}_{l}w^{t}{\left(w^{t}\right)}^{H}{\tilde{H}}_{l}^{H}}{y_{l}^{t}\left(y_{l}^{t}+{\left|x_{l}^{t}\right|}^{2}\right)}\\ & +\frac{\sum_{i=1}^{L}{\left(a_{i}^{t}\right)}^{2}{\tilde{G}}_{l}w^{t}{\left(w^{t}\right)}^{H}{\tilde{G}}_{l}^{H}}{1+z_{l}^{t}}\forall l\\ \varphi_{l} & =\sum_{i=1,i\neq l}^{L}{\tilde{G}}_{l}w^{t}{\left(w^{t}\right)}^{H}{\tilde{G}}_{l}^{H}{\hat{\theta }}^{t}{\left(a_{i}^{t}\right)}^{2}+\frac{{\tilde{H}}_{l}w^{t}{\left(w^{t}\right)}^{H}{\tilde{H}}_{l}^{H}{\hat{\theta }}^{t}{\left(a_{l}^{t}\right)}^{2}}{y_{l}^{t}}\forall l \end{array}$$

The unit modulus constraint (10d) makes the problem difficult to solve. Although the semidefinite relaxation (SDR) method can be used to solve it, it is time consuming [11]. We provide two efficient algorithms to tackle this problem. We start by introducing the MM method first.

(1) The MM method: the main idea behind the MM method is to transform the problem (28) into a series of tractable approximated subproblems [24]. Generally, the MM method contains two steps. In the majorization step, we construct a surrogate function that upperbounds the objective function of (28) up to a constant. Let us denote the objective function of (28) as $f\left(\hat{\theta }\right)$. According to the second order Taylor expansion of $f\left(\hat{\theta }\right)$ at a fixed point ${\hat{\theta }}^{k}$, we obtain the following inequality:

(30) $$\begin{array}{cc} f\left(\hat{\theta }\right) & \leq \lambda_{\max}{\hat{\theta }}^{H}\hat{\theta }-2ℜ\left\{{\hat{\theta }}^{H}\left[\left(\Omega -\lambda_{\max}I_{M\times M}\right){\hat{\theta }}^{k}+\varphi \right]\right\}\\ & +{\left({\hat{\theta }}^{k}\right)}^{H}\left(\Omega -\lambda_{\max}I_{M\times M}\right){\hat{\theta }}^{k} \end{array}$$

where $\lambda_{\max}$ is the maximum eigenvalue of matrix Ω. By taking the unit modulus constraint $\left|{\hat{\theta }}_{m}\right|=1,\forall m$ into consideration, we have ${\hat{\theta }}^{H}\hat{\theta }=M$. Then, Equation (30) can be transformed as

(31) $$\begin{array}{cc} f\left(\hat{\theta }\right) & \leq \lambda_{\max}M-2ℜ\left\{{\hat{\theta }}^{H}\left[\left(\Omega -\lambda_{\max}I_{M\times M}\right){\hat{\theta }}^{k}+\varphi \right]\right\}\\ & +{\left({\hat{\theta }}^{k}\right)}^{H}\left(\Omega -\lambda_{\max}I_{M\times M}\right){\hat{\theta }}^{k}\overset{\Delta }{\;=}g\left(\hat{\theta }\left|{\hat{\theta }}^{k}\right.\right) \end{array}$$

where $g\left(\hat{\theta }\left|{\hat{\theta }}^{k}\right.\right)$ is the surrogate function of $f\left(\hat{\theta }\right)$ at point ${\hat{\theta }}^{k}$. In the minimization step, we derive the corresponding solution by minimizing $g\left(\hat{\theta }\left|{\hat{\theta }}^{k}\right.\right)$ and solve problem (28) iteratively. By omitting the constants, the subproblem of minimizing $g\left(\hat{\theta }\left|{\hat{\theta }}^{k}\right.\right)$ at the *k*th iteration can be rewritten as

(32a) $$\begin{array}{cc} \min_{\hat{\theta }} & -2ℜ\left\{{\hat{\theta }}^{H}q^{k}\right\} \end{array}$$

(32b) $$\begin{array}{cc} & s.t.(10d) \end{array}$$

where $q^{k}=\left(\Omega -\lambda_{\max}I_{M\times M}\right){\hat{\theta }}^{k}+\varphi$. It is easy to see that the problem (32) has a closed-form solution, which is derived as

(33) $${\hat{\theta }}^{k+1}=\exp\left(j\arg\left[q^{k}\right]\right)$$

According to the principle of the MM method, the proposed MM algorithm is described in Algorithm 3.
**Algorithm 3** The MM algorithm for problem (28)
1: **Initialization:** set the accuracy ε, the maximum iteration number *K*, and a feasible point $\left\{{\left\{a_{l}^{0}\right\}}_{l=1}^{L},w^{0},{\hat{\theta }}^{0}\right\}$;
2: **Repeat**
*k*
3: **Calculate**
$q^{k}=\left(\Omega -\lambda_{\max}I_{M\times M}\right){\hat{\theta }}^{k}+\varphi$;
4: **Update** ${\hat{\theta }}^{k+1}$ according to (33);
5: **Until**
fθ^k+1−fθ^kfθ^k+1−fθ^kfθ^k+1fθ^k+1≤ε or k>K;
6: **Output**
${\hat{\theta }}^{*}$.


(2) The ADMM method: Note that problem (28) has a similar structure to problem (22) except for the modulus constraint $\left|\theta_{m}\right|=1,\forall m$. Therefore, after making some modifications to (27), we obtain the following expressions:

(34) $${\left[{\hat{\theta }}^{k+1}\right]}_{m}=\left\{\begin{array}{cc} \frac{{\left[r_{\theta }^{k+1}-\rho_{\theta }^{-1}p_{\theta }^{k}\right]}_{m}}{\left|{\left[r_{\theta }^{k+1}-\rho_{\theta }^{-1}p_{\theta }^{k}\right]}_{m}\right|}, & \mathrm{if}{\left[r_{\theta }^{k+1}-\rho_{\theta }^{-1}p_{\theta }^{k}\right]}_{m}\neq 0\\ {\left[{\hat{\theta }}^{k}\right]}_{m}, & \mathrm{if}{\left[r_{\theta }^{k+1}-\rho_{\theta }^{-1}p_{\theta }^{k}\right]}_{m}=0 \end{array}\right.$$

where $r_{\theta }$, $\rho_{\theta }$, and $p_{\theta }$ are corresponding auxiliary variables related to $\hat{\theta }$. A closed-form solution of problem (28) can be obtained by adopting the ADMM method as described in Algorithm 2 in the preceding section, which is omitted here for brevity.

Lastly, the WSSR maximization problem (10) was decomposed into three solvable subproblems that were solved iteratively by corresponding methodsṪhe proposed AO algorithm is summarized in Algorithm 4.
**Algorithm 4** The proposed AO algorithm for the WSSR maximization problem
1: **Initialization:** set the maximum iteration number *K*, the accuracy ε, and a feasible point $\left\{{\left\{a_{l}^{0}\right\}}_{l=1}^{L},w^{0},{\hat{\theta }}^{0}\right\}$;
2: **Repeat**
*k*
3: **Obtain**
${\left\{a_{l}^{k+1}\right\}}_{l=1}^{L}$ via solving (15) with fixed $\left\{{\left\{a_{l}^{k}\right\}}_{l=1}^{L},w^{k},{\hat{\theta }}^{k}\right\}$;
4: **Obtain**$w^{k+1}$ via solving (20) using the ADMM method with fixed $\left\{{\left\{a_{l}^{k+1}\right\}}_{l=1}^{L},w^{k},{\hat{\theta }}^{k}\right\}$;
5: **Obtain**
${\hat{\theta }}^{k+1}$ via solving (28) using the ADMM or MM method with fixed $\left\{{\left\{a_{l}^{k+1}\right\}}_{l=1}^{L},w^{k+1},{\hat{\theta }}^{k}\right\}$;
6: **Until** $R_{s}^{k+1}-R_{s}^{k}<\varepsilon$ or k>K;
7: **Output** $\left\{{\left\{a_{l}^{*}\right\}}_{l=1}^{L},w^{*},{\hat{\theta }}^{*}\right\}$.


### 3.5. Complexity Analysis

In this part, we analyze the computational complexity of the proposed algorithms. We first denote the iteration number of the ADMM, MM, and AO algorithms as $T_{ADMM}$, $T_{MM}$, and $T_{AO}$, respectively. Specifically, the complexity of the proposed ADMM method mainly lies in updating r, the complexity of which is $O\left(N^{2}+N^{3}\right)$, where $O\left(N^{3}\right)$ and and $O\left(N^{2}\right)$, respectively, denote the complexity for calculating ${\left(\rho I+2A\right)}^{-1}$ and other multiplication operations in updating r. We note that the inverse matrix ${\left(\rho I+2A\right)}^{-1}$ is calculated only once during the whole algorithm. Therefore, the total complexity of the ADMM method is $O\left(T_{ADMM}N^{2}+N^{3}\right)$ [25]. Similar to the ADMM algorithm, the complexity of the MM algorithm mainly lies in two parts. At the beginning of the MM algorithm, calculating $\lambda_{\max}$ incurs $O\left(M^{3}\right)$ computational complexity. In each iteration of the MM algorithm, the main complexity comes from updating $q^{k}$, the complexity of which is $O\left(M^{2}\right)$. Thus, the complexity of the MM algorithm is $O\left(T_{MM}M^{2}+M^{3}\right)$ [26]. It can be observed that the complexity of the ADMM and MM methods mainly depends on the iteration times, which is typically several hundreds of seconds. Lastly, the overall complexity of the proposed AO algorithm is $O\left(T_{AO}\left(\left(T_{ADMM}N^{2}+N^{3}\right)+\left(T_{ADMM/MM}M^{2}+M^{3}\right)\right)\right)$.

## 4. Simulation Results

**Figure 2:**
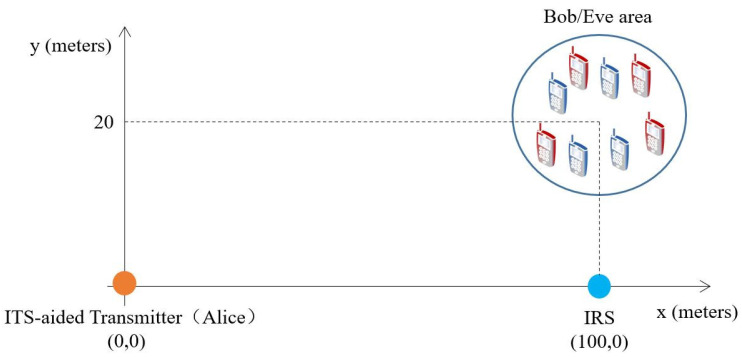
Simulation setup.

In this section, we evaluate the performance of our proposed algorithms by numerical simulation results. The simulation scenario for the coordinates (in meters) is illustrated in Figure 2, where Alice and the IRS are located at (0, 0) and (100, 0), respectively. *L* Bobs are randomly scattered in a circle centered at (100, 20) with a radius of 5 m. Each Bob is eavesdropped by one Eve, which randomly located within a circle centered at the Bob with radius 2 m. The heights of Alice, IRS, Bobs, and Eves are set as 20 m, 10 m, 1.5 m and 1.5 m, respectively. The large-scale path loss is modeled as $\mathrm{PL}=PL_{0}-10\log_{10}\left(d/d_{0}\right)$ dB, where $PL_{0}$ is the path-loss at the reference distance and *d* is the link distance in meters. The path loss exponents for the Alice-Bobs/Eves link, the Alice-IRS link, and the IRS–Bobs/Eves link are set as $\alpha_{t}=4$, $\alpha_{tr}=2$ and $\alpha_{r}=2$, respectively. Other parameters, unless otherwise specified, are set as follows: N=48,M=64, $P_{max}=10\;\mathrm{dBm}$, $\sigma_{d,l}^{2}=\sigma_{e,l}^{2}=-70\;\mathrm{dBm}$, $d_{0}=1\;m$, L=4, and Ricean factor κ=3.

**Figure 3:**
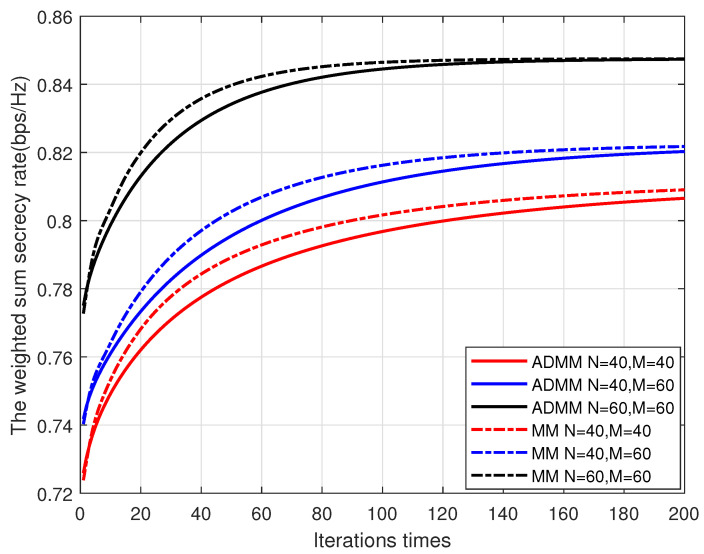
The convergence performance of ADMM and MM algorithms.

We first study the convergence behaviors of the proposed algorithm with different numbers of transmissive element *N* and reflective element *M*. Figure 3 shows the convergence properties of ADMM and MM algorithms for optimizing the phase shift of IRS in the first AO iteration. It can be observed that the WSSR achieved by both methods increases with the iteration number. Although the ADMM method converges a little more slowly than the MM method, they both tend to converge within 200 iterations, which verifies the convergence of the two methods.

**Figure 4:**
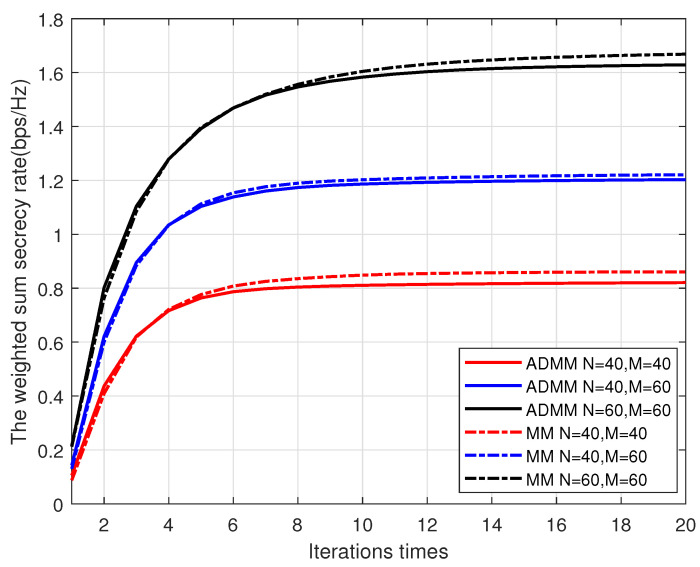
The convergence behavior of OA algorithms.

The convergence performance of the AO algorithm is shown in Figure 4 for different *N* and *M*. It is clear that the AO algorithm tends to converge within 15 iterations for all *N* and *M* combinations considered, which demonstrates the convergence of the suggested AO algorithms. Moreover, the increase in *N* or *M* leads to higher WSSR but slower convergence performance, which is because large *N* or *M* indicates more variables that need to be optimized. Also, given the same *N* and *M* combinations, the AO-MM and AO-ADMM algorithms have a similar convergence speed, but the WSSR obtained by the AO-MM method is higher than that of the AO-ADMM method.

Next, we evaluate the performance of the proposed algorithm and contrast it with the benchmark methodologies shown below: (1) Random IRS: Only ${\left\{a_{l}\right\}}_{l=1}^{L}$ and w are optimized, and the phase for each IRS reflective components is generated uniformly and independently over [0,2π). (2) No IRS: Replace the IRS-related channel matrices by zero matrices and remove step 5 from Algorithm 4. (3) Equal power: Allocate the same power to each Bob, i.e., set $a_{l}=P_{max}/L\;\forall l$. (4) The upper bound: Obtaining the optimal value of power allocation, ITS beamforming, and IRS phase shift numerically by using CVX toolbox. Specifically, ${\left\{a_{l}^{*}\right\}}_{l=1}^{L}$ and $w^{*}$ are obtained by directly solving problem (15) and (20) as QCQP and QP, respectively. ${\hat{\theta }}^{*}$ is obtained by solving problem (28) via applying the SDR method and Gaussian randomization techniques.

**Figure 5:**
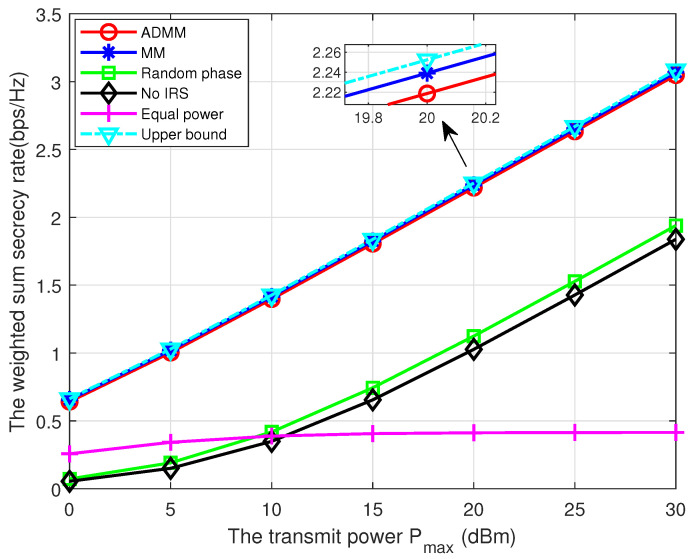
The WSSR versus the transmit power.

Figure 5 compares the WSSR performance versus the transmit power $P_{max}$ of all schemes. We can see that the WSSR increases with $P_{max}$. Moreover, both ADMM and MM algorithms achieve near-optimal performance compared to the upper bound and also significantly outperform other schemes, demonstrating the performance of our proposed transmission strategy. Further, the random phase scheme outperforms the no IRS scheme, which verifies the benefits of utilizing IRS. Lastly, the equal power scheme achieves the worst performance when $P_{max}>10\;\mathrm{dBm}$, which can be explained by the fact that the inter-user interference becomes the primary performance constraint in the high transmit power region.

**Figure 6:**
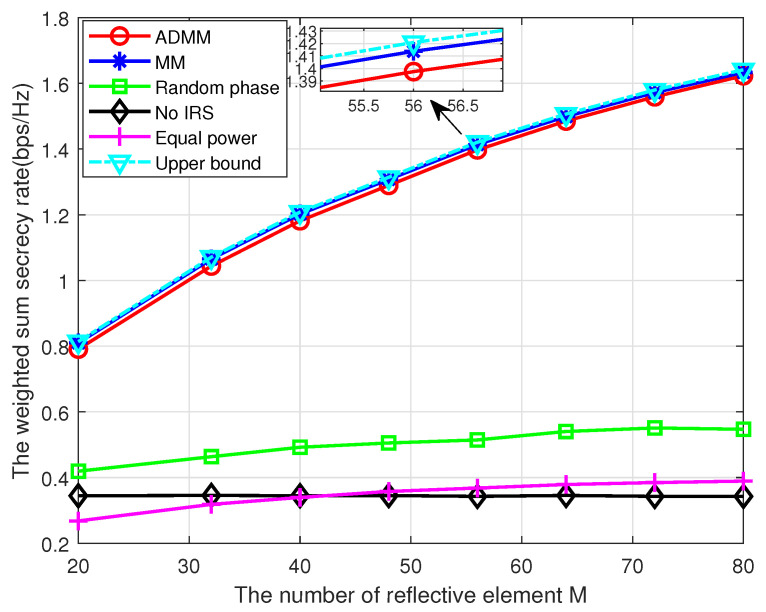
The WSSR versus the reflective element number.

Then, we study the impact of the number of IRS reflective elements *M* on the WSSR of a different scheme in Figure 6. We can see that the WSSR experiences a great increase with *M* for both ADMM and MM schemes, which indicates that increasing the number of IRS elements can improve the WSSR effectively. By contrast, the WSSR of the random phase scheme is significantly lower that of the ADMM or MM schemes, which verifies the advantage of our proposed algorithm. Moreover, the WSSR of random phase scheme is higher than that of the no IRS case, and the gap only increases slightly with *M*, which is due to the fact that more signals can be reflected by IRS with larger *M* and these additional signals can enhance secrecy performance only if the phase shifter been properly designed.

**Figure 7:**
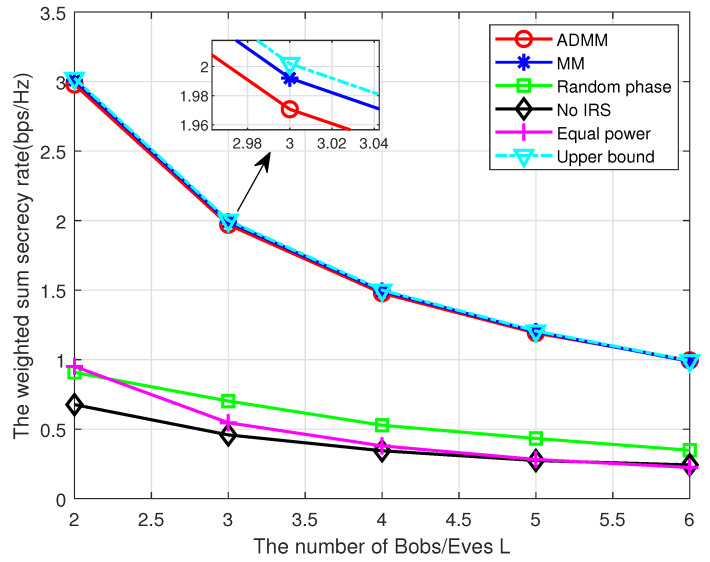
TheWSSR versus L.

Next, we investigate the system performance with a different number of Bobs/Eves *L*, as shown in Figure 7. As we can see, the WSSR declines with *L*, which is because the inter-user interference increases with *L* and the weight *1/L* for each Bobs decreases with *L*. As a result, the WSSR tends to decrease.

**Figure 8:**
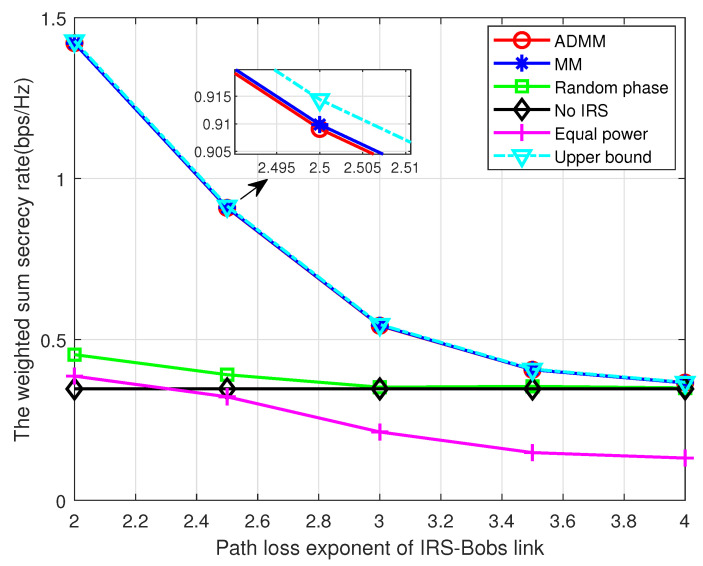
The WSSR versus pathloss exponent of the IRS–Bobs link.

**Figure 9:**
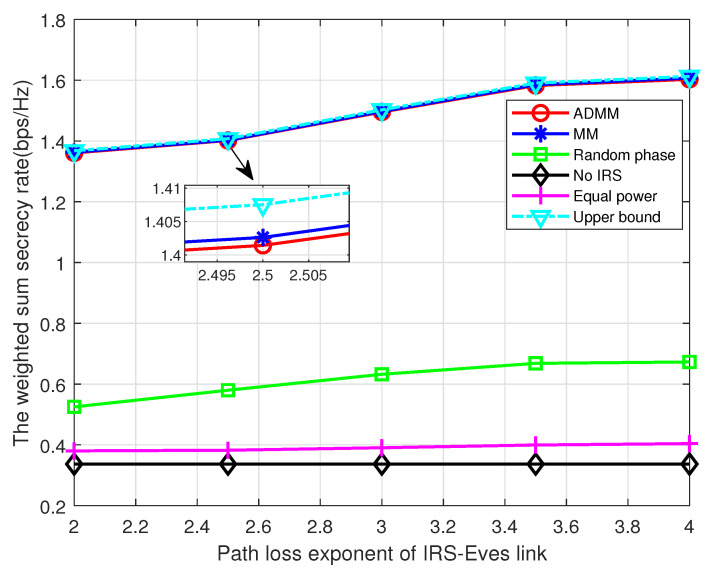
The WSSR versus pathloss exponent of the IRS–Eves link.

Lastly, we discuss the relationship between system performance and path loss exponent. As Figure 8 shows, the WSSR of the proposed algorithms decreases dramatically with the increase in the path loss exponent of the IRS–Bobs link. This is mainly because more severe path loss will decrease the power of the reflected signal from the IRS, which jeopardizes the system performance. Meanwhile, the increase in the path loss exponent of the IRS–Eves link improves the WSSR as depicted in Figure 9, which is due to the fact that more severe large-scale fading degrades the reflected signal at Eve. These two figures demonstrate a technical insight that, in order to achieve a better performance, the IRS should be carefully installed so that there are fewer obstructions in the legitimate link or more obstructions in the eavesdropping link.

## 5. Conclusions

This paper has investigated an ITS-and IRS-empowered MU-MISOME secure communication network where an IRS is deployed to create a programmable wireless environment, and an ITS-based transmitter is adopted to perform energy-efficient beamforming. To be specific, we have maximized the WSSR by jointly optimizing the power allocation, ITS beamforming, and phase shift of IRS while guaranteeing transmit power constraint and unit-modulus constraints. The non-concave objective function was transformed into a tractable form by using the SCA technique. An efficient AO scheme was developed to convert the reformulated problem into three solvable subproblems. The KKT conditions and the ADMM and MM methods were adopted to derived the closed-form solution for each subproblem. The numerical results demonstrated that the proposed schemes can achieve near-optimal performance and the IRS can improve the system WSSR effectively.

The performance of the MM algorithm is slightly better than that of the ADMM algorithm, while the ADMM algorithm is a more general approach since it does not require the objective function to be differentiable. Both secrecy transmission schemes we proposed are suitable for resource-constrained IoT devices because they do not rely on computational complexity. In addition, the IRS can be flexibly deployed in IoT networks without causing additional interference or changing the network topology.

## Figures and Tables

**Table 1:** List of main notations in the considered system.

Symbol	Definition
*N*	number of ITS elements
*M*	number of IRS elements
$F\in C^{M\times N}$	channel matrix from ITS to IRS
$h_{d,l}\in C^{N\times 1}$/$g_{d,l}\in C^{N\times 1}$	channel vector from ITS to the *l*th Bob/Eve
$h_{r,l}\in C^{M\times 1}$/$g_{r,l}\in C^{M\times 1}$	channel vector from IRS to the *l*th Bob/Eve
$w\in C^{N\times 1}$	beamforming vector of ITS
$\theta \in C^{M\times 1}$	phase shift vector of IRS
$s_{l}\in C$	confidential message for the *l*th Bob
$a_{l}>0$	power allocation factor for $s_{l}$
$n_{d,l}$/$n_{e,l}$	AWGN at *l*th Bob/Eve

## Data Availability

Data available on request from the authors.

## Appendix A

**Proof** **of** (12) **and** (13). The prove of (12) is straightforward since $R_{d,l}^{lb}$ can be obtained directly by using Lemma 1 after rewriting $R_{d,l}$ as follows: ln1+xl2xl2ylyl. Next, let us derive $R_{e,l}^{ub}$. We first rewrite $R_{e,l}$ as

(A1) $$\begin{array}{c} R_{e,l}=\ln\left(1+z_{l}\right)-\ln\left(1+\sum_{i=1,i\neq l}^{L}{\left|{\hat{\theta }}^{H}{\tilde{G}}_{l}wa_{i}\right|}^{2}\right) \end{array}$$

According to the first-order condition of the concave function, an upper bound of $\ln\left(1+z_{l}\right)$ can be derived as follows:

(A2) $$\begin{array}{c} \ln\left(1+z_{l}\right)\leq \ln\left(1+z_{l}^{t}\right)+\frac{1+z_{l}}{1+z_{l}^{t}}-1 \end{array}$$

For (11), let $v=v^{t}=1$; then, (11) can be written as follows:

(A3) $$\ln\left(1+{\left|u\right|}^{2}\right)\geq \ln\left(1+{\left|u^{t}\right|}^{2}\right)-{\left|u^{t}\right|}^{2}+2ℜ\left\{{\left(u^{t}\right)}^{*}u\right\}-\frac{{\left|u^{t}\right|}^{2}\left(1+{\left|u\right|}^{2}\right)}{1+{\left|u^{t}\right|}^{2}}$$

According to (A3), the following inequality can be derived by keeping $u_{i}$ fixed for ∀i≠l,

(A4) $$\begin{array}{cc} \; & \ln\left(1+\sum_{i=1,i\neq l}^{L}{\left|u_{i}\right|}^{2}+{\left|u_{l}\right|}^{2}\right)\geq \ln\left(1+\sum_{i=1,i\neq l}^{L}{\left|u_{i}^{t}\right|}^{2}+{\left|u_{l}^{t}\right|}^{2}\right)\\ & -{\left|u_{l}^{t}\right|}^{2}+2ℜ\left\{{\left(u_{l}^{t}\right)}^{*}u_{l}\right\}-\frac{{\left|u_{l}^{t}\right|}^{2}\left(1+\sum_{i=1,i\neq l}^{L}{\left|u_{i}\right|}^{2}+{\left|u_{l}\right|}^{2}\right)}{1+\sum_{i=1,i\neq l}^{L}{\left|u_{i}^{t}\right|}^{2}+{\left|u_{l}^{t}\right|}^{2}} \end{array}$$

Using (A4) with l=1,…,L, we derive the following inequality:

(A5) $$\begin{array}{cc} \; & \ln\left(1+\sum_{i=1}^{L}{\left|u_{i}\right|}^{2}\right)\geq \ln\left(1+\sum_{i=1,}^{L}{\left|u_{l}^{t}\right|}^{2}\right)-\sum_{i=1}^{L}{\left|u_{l}^{t}\right|}^{2}\\ & +\sum_{i=1}^{L}2ℜ\left\{{\left(u_{l}^{t}\right)}^{*}u_{l}\right\}-\frac{\sum_{i=1}^{L}{\left|u_{l}^{t}\right|}^{2}\left(1+\sum_{i=1}^{L}{\left|u_{i}\right|}^{2}\right)}{\left(1+\sum_{i=1}^{L}{\left|u_{l}^{t}\right|}^{2}\right)} \end{array}$$

According to (A5), we have

(A6) $$\begin{array}{cc} \; & \ln\left(1+\sum_{i=1,i\neq l}^{L}{\left|{\hat{\theta }}^{H}{\tilde{G}}_{l}wa_{i}\right|}^{2}\right)\geq \ln\left(1+c_{l}^{t}\right)-c_{l}^{t}\\ & +2\sum_{i=1,i\neq l}^{L}ℜ\left\{a_{i}{\left(w\right)}^{H}{\tilde{G}}_{l}^{H}\hat{\theta }{\left({\hat{\theta }}^{t}\right)}^{H}{\tilde{G}}_{l}w^{t}a_{i}^{t}\right\}\\ & -\frac{c_{l}^{t}}{1+c_{l}^{t}}\left(1+\sum_{i=1,i\neq l}^{L}{\left|{\hat{\theta }}^{H}{\tilde{G}}_{l}wa_{i}\right|}^{2}\right) \end{array}$$

Finally, it is straightforward to obtain $R_{e,l}^{ub}$ by substituting (A2) and (A6) back into (A1). □

## References

[1] Shannon C.E. (1949). Communication theory of secrecy systems. Bell Syst. Tech. J..

[2] Wyner A.D. (1975). The wire-tap channel. Bell Syst. Tech. J..

[3] Csiszár I., Korner J. (1978). Broadcast channels with confidential messages. IEEE Trans. Inf. Theory.

[4] Yamamoto H. (1991). A coding theorem for secret sharing communication systems with two gaussian wiretap channels. IEEE Trans. Inf. Theory.

[5] Fakoorian S.A.A., Swindlehurst A.L. Optimal power allocation for gsvd-based beamforming in the mimo gaussian wiretap channel. Proceedings of the 2012 IEEE International Symposium on Information Theory Proceedings.

[6] Chen X., Ng D.W.K., Gerstacker W.H., Chen H.-H. (2017). A survey on multiple-antenna techniques for physical layer security. IEEE Commun. Surv. Tutorials.

[7] Cui M., Zhang G., Zhang R. (2019). Secure wireless communication via intelligent reflecting surface. IEEE Wirel. Commun. Lett..

[8] Jiang W., Han B., Habibi M.A., Schotten H.D. (2021). The road towards 6g: A comprehensive survey. IEEE Open J. Commun. Soc..

[9] Wu Q., Zhang R. Beamforming optimization for intelligent reflecting surface with discrete phase shifts. Proceedings of the ICASSP 2019—2019 IEEE International Conference on Acoustics, Speech and Signal Processing (ICASSP).

[10] Chu Z., Zhu Z., Zhou F., Zhang M., Al-Dhahir N. (2021). Intelligent reflecting surface assisted wireless powered sensor networks for internet of things. IEEE Trans. Commun..

[11] Chu Z., Hao W., Xiao P., Shi J. (2020). Intelligent reflecting surface aided multi-antenna secure transmission. Prog. Artif. Intell..

[12] Chu Z., Hao W., Xiao P., Mi D., Liu Z., Khalily M., Kelly J.R., Feresidis A.P. (2020). Secrecy rate optimization for intelligent reflecting surface assisted MIMO system. IEEE Trans. Inf. Forensics Secur..

[13] Niu H., Chu Z., Zhou F., Zhu Z., Zhen L., Wong K.-K. (2022). Robust design for intelligent reflecting surface-assisted secrecy SWIPT network. IEEE Trans. Wirel. Commun..

[14] Ren H., Liu X., Pan C., Peng Z., Wang J. (2022). Performance analysis for RIS-aided secure massive MIMO systems with statistical csi. IEEE Wirel. Commun. Lett..

[15] Yang Z., Xu W., Huang C., Shi J., Shikh-Bahaei M. (2021). Beamforming design for multiuser transmission through reconfigurable intelligent surface. IEEE Trans. Commun..

[16] Li Z., Chen W., He C., Bai X., Lu J. (2022). Multi-antenna systems by transmissive reconfigurable meta-surface. arXiv.

[17] Li Z., Chen W., Cao H. (2022). Beamforming design and power allocation for transmissive RMS-based transmitter architectures. IEEE Wirel. Commun. Lett..

[18] Li Z., Chen W., Zhang Z., Wu Q., Cao H., Li J. (2023). Robust sum-rate maximization in transmissive RMS transceiver-enabled SWIPT networks. IEEE Internet Things J..

[19] Niu H., Lin Z., Chu Z., Zhu Z., Xiao P., Nguyen H.X., Lee I., Al-Dhahir N. (2023). Joint beamforming design for secure RIS-assisted IoT networks. IEEE Internet Things J..

[20] Wu Q., Zhang R. Intelligent reflecting surface enhanced wireless network: Joint active and passive beamforming design. Proceedings of the 2018 IEEE Global Communications Conference (GLOBECOM).

[21] Nasir A.A., Tuan H.D., Duong T.Q., Poor H.V. (2017). Secrecy rate beamforming for multicell networks with information and energy harvesting. IEEE Trans. Signal Process..

[22] Grant M., Boyd S.P. (2014). CVX: Matlab Software for Disciplined Convex Programming. http://cvxr.com/cvx.

[23] Niu H., Chu Z., Zhou F., Zhu Z., Zhang M., Wong K.-K. (2021). Weighted sum secrecy rate maximization using intelligent reflecting surface. IEEE Trans. Commun..

[24] Ying S., Babu P., Palomar D.P. (2016). majorization–minimization algorithms in signal processing, communications, and machine learning. IEEE Trans. Signal Process..

[25] Li Q., Li C., Lin J. (2019). Constant modulus secure beamforming for multicast massive MIMO wiretap channels. IEEE Trans. Inf. Forensics Secur..

[26] Jiang W., Chen B., Zhao J., Xiong Z., Ding Z. (2021). Joint Active and Passive Beamforming Design for the IRS-Assisted MIMOME-OFDM Secure Communications. IEEE Trans. Veh. Technol..

